# Toll-like receptor 1 as a possible target in non-alcoholic fatty liver disease

**DOI:** 10.1038/s41598-021-97346-9

**Published:** 2021-09-08

**Authors:** Anja Baumann, Anika Nier, Angélica Hernández-Arriaga, Annette Brandt, Maria J. Lorenzo Pisarello, Cheng J. Jin, Esther Pilar, Amélia Camarinha-Silva, Jörn M. Schattenberg, Ina Bergheim

**Affiliations:** 1 Department of Nutritional Sciences, Molecular Nutritional Science, University of Vienna, Althanstraße 14 (UZA2), 1090 Vienna, Austria; 2 Institute of Animal Science, University of Hohenheim, Stuttgart, Germany; 3 Institute of Nutrition, SD Model Systems of Molecular Nutrition, Friedrich-Schiller-University Jena, Jena, Germany; 4 Metabolic Liver Research Program, Department of Internal Medicine I, University Medical Center of the Johannes Gutenberg-University, Mainz, Germany

**Keywords:** Hepatology, Liver diseases, Non-alcoholic fatty liver disease

## Abstract

Toll-like receptors (TLRs) in the liver compartment have repeatedly been attributed to the development of non-alcoholic fatty liver disease (NAFLD). Knowledge on TLR expression in blood cells and their relation to intestinal microbiota and NAFLD development is limited. Here, we determined TLR expression patterns in peripheral blood mononuclear cells (PBMCs) of NAFLD patients and controls, their relation to intestinal microbiota and the impact of TLRs found altered in NAFLD development. Markers of intestinal permeability in blood and *TLR* mRNA expression in PBMCs were determined in 37 NAFLD patients and 15 age-matched healthy controls. Fecal microbiota composition was evaluated in 21 NAFLD patients and 9 controls using 16S rRNA gene amplicon sequencing. Furthermore, *TLR1*^−/−^ and C57BL/6 mice (n = 5–6/group) were pair-fed a liquid control or a fat-, fructose- and cholesterol-rich diet. Intestinal microbiota composition and markers of intestinal permeability like zonulin and bacterial endotoxin differed significantly between groups with the latter markers being significantly higher in NAFLD patients. Expression of *TLR1-8* and *10* mRNA was detectable in PBMCs; however, only *TLR1* expression, being higher in NAFLD patients, were significantly positively correlated with the prevalence of *Holdemanella* genus while negative correlations were found with *Gemmiger* and *Ruminococcus* genera. *TLR1*^−/−^ mice were significantly protected from the development of diet-induced NAFLD when compared to wild-type mice. While intestinal microbiota composition and permeability differed significantly between NAFLD patients and healthy subjects, in PBMCs, only *TLR1* expression differed between groups. Still, targeting these alterations might be a beneficial approach in the treatment of NAFLD in some patients.

## Introduction

Non-alcoholic fatty liver disease (NAFLD) is by now one of the most common liver diseases worldwide affecting ~ 25% of the global general population with prevalence still increasing^1,2^. Accordingly, the demand for therapeutic interventions to treat patients at risk for the development of end-stage liver disease is tremendous and many have failed (for overview see^3^). As NAFLD is a disease slowly progressing along a spectrum ranging from hepatic steatosis defined as non-alcoholic fatty liver to inflammatory non-alcoholic steatohepatitis (NASH) and finally end-stage liver disease including cirrhosis and hepatocellular carcinoma^4^, opportunity to provide care to these patients seems to be large. However, as molecular mechanisms involved in the onset as well as the progression of the disease are still not fully understood, universally accepted strategies for prevention and therapy still mainly focus on life-style interventions, being often afflicted with low compliance and high relapse rates while drug-based therapies are still limited.

Toll-like receptors (TLRs) are evolutionarily conserved type I glycoprotein transmembrane receptors that posse a leucine-rich repeat in their ectodomains to recognize pathogen-associated molecular patterns (PAMP)^5^. Upon PAMP binding, conformation is changed leading to the formation of homo- or heterodimers, and subsequently, actions of cell signaling either involving an activation of myeloid differentiation primary response 88 (Myd88)- or TIR-domain-containing adapter-inducing interferon β (TRIF)/TRIF-related adaptor molecule (TRAM)-dependent signaling cascades^6^. TLRs are activated by a variety of bacterial, viral, fungal and parasitic antigens, but also endogenous ligands with each TLR responding to an unique subset of triggers^7^. In humans, 10 TLRs are expressed of which all have been detected in liver tissue^8,9^. Furthermore, studies in NAFLD patients and mice with diet-induced NAFLD suggest that expressions of TLRs in liver tissue are altered in settings of NAFLD being associated with intestinal barrier dysfunction and increased bacterial toxin levels^9,10^. Furthermore, many clinical studies indicate that patients with NAFLD exhibit a reduced bacterial diversity, sometimes also referred to as `gut dysbiosis´ (for overview see^11^). Indeed, studies suggest that an altered intestinal microbiota could lead to hepatic fat accumulation through several mechanisms including an increased translocation of bacterial toxins promoting low-grade inflammation and changes in gut permeability (for overview see^12^). These and other observations contribute to the concept of metabolic inflammation as an underlying modification that leads to the liver phenotype, but is also linked to extrahepatic morbidity in NAFLD (for overview see^13^).

However, whether similar changes in TLR expressions are also found in other tissues, like blood cells, and if these are related to alterations of intestinal microbiota composition and barrier function, and moreover, are indicative of disease targets have not been studied in-depth. Starting from this background, the first aim of the present study was to assess *TLR* expression patterns in peripheral blood mononuclear cells (PBMCs) derived from NAFLD patients and age-matched controls and their relation to intestinal microbiota composition and barrier function. In a second step, the impact of differences found regarding *TLR1* expression between NAFLD patients and controls on disease development was assessed in a dietary mouse model employing *TLR1*^−/−^ mice.

## Results

### Characteristics of NAFLD patients and controls

Age was similar between NAFLD patients and controls (Table 1), while gender differed by trend between groups (*p* = 0.0635)*.* The mean value of steatosis stage of NAFLD patients was 1.7 and was based on the score. NAFLD patients suffered from a hepatic steatosis ranging from mild to moderate. Activity of transaminases and gamma glutamyl transferase (γ-GT) in plasma but also markers of glucose and lipid metabolism as well as levels of C-reactive protein (CRP), plasminogen activator inhibitor 1 (PAI-1), uric acid, leptin and body mass index (BMI) as well as waist circumference were significantly higher in NAFLD patients than in controls. Adiponectin levels were significantly lower in NAFLD patients than in controls. Leptin to adiponectin ratio, shown to be indicative of visceral adiposity^14^, was also significantly higher in NAFLD patients than in controls (Table 1). Furthermore, both, systolic and diastolic blood pressure were significantly higher in patients with NAFLD than in controls (Table 1) with 70% of the NAFLD patients suffering from hypertension. 14% of patients had diabetes type 2 (fasting blood glucose levels ≥ 7.0 mmol/l) and 64% had a metabolic syndrome as defined by the WHO/International Diabetes Federation diagnostic criteria (WHO/IDF, 2006).

**Table 1:** Characteristics of study participants.

	C	NAFLD
n	15	37
Sex (m/f)	5/10	24/13
Age	46.2 ± 1.6	47.7 ± 2.2
BMI (kg/m^2^)	23.6 ± 0.7	31.6 ± 1.0*
Waist circumference in cm (m/f)	86 ± 2/73 ± 2	111 ± 3*/105 ± 5*
Steatosis (stage)	0 ± 0	1.7 ± 0.1*
ALT (U/l)	19.1 ± 1.6	67.0 ± 7.0*
AST (U/l)	20.1 ± 2.1	41.0 ± 2.8*
γ-GT (U/l)	17.9 ± 2.2	102.9 ± 15.8*
Systolic blood pressure (mmHg)	125.5 ± 2.7	140.1 ± 3.2*
Diastolic blood pressure (mmHg)	80.9 ± 2.0	89.8 ± 1.6*
Triglycerides (mg/dl)	83.5 ± 15.4	160.9 ± 15.2*
Total cholesterol (mg/dl)	189.4 ± 9.1	219.8 ± 6.7*
LDL (mg/dl)	103.3 ± 7.4	136.5 ± 6.3*
HDL in mg/dl (m/f)	60.4 ± 4.3/76.9 ± 3.9	45.5 ± 2.9*/61.6 ± 5.5*
Insulin (µU/ml)	4.2 ± 0.2	14.6 ± 1.9*
Fasting glucose (mg/dl)	80.3 ± 2.8	103.0 ± 4.3*
HOMA-IR	0.9 ± 0.1	4.2 ± 1.0*
Leptin (ng/ml)	4.4 ± 0.7	12.1 ± 1.6*
Adiponectin (ng/ml)	53.2 ± 6.8	24.0 ± 2.9*
Leptin/adiponectin	0.33 ± 0.2	0.74 ± 0.2*
PAI-1 (U/l)	10.6 ± 2.2	45.0 ± 3.8*
CRP (mg/l)	1.6 ± 0.5	5.0 ± 1.2*
Uric acid (mg/dl)	4.2 ± 0.3	6.2 ± 0.3*

Data are shown as total numbers and means ± SEM, respectively, n = 14–15 controls and n = 32–37 NAFLD patients, *p ≤ 0.05 compared to controls.

*γ-GT* gamma glutamyl transferase, *ALT* alanine aminotransferase, *AST* aspartate aminotransferase, *BMI* body mass index, *C* healthy controls, *CRP* C-reactive protein, *NAFLD* patients with non-alcoholic fatty liver disease, *PAI-1* plasminogen activator inhibitor 1.

### Fecal microbiota in patients with NAFLD and controls

**Figure 1:**
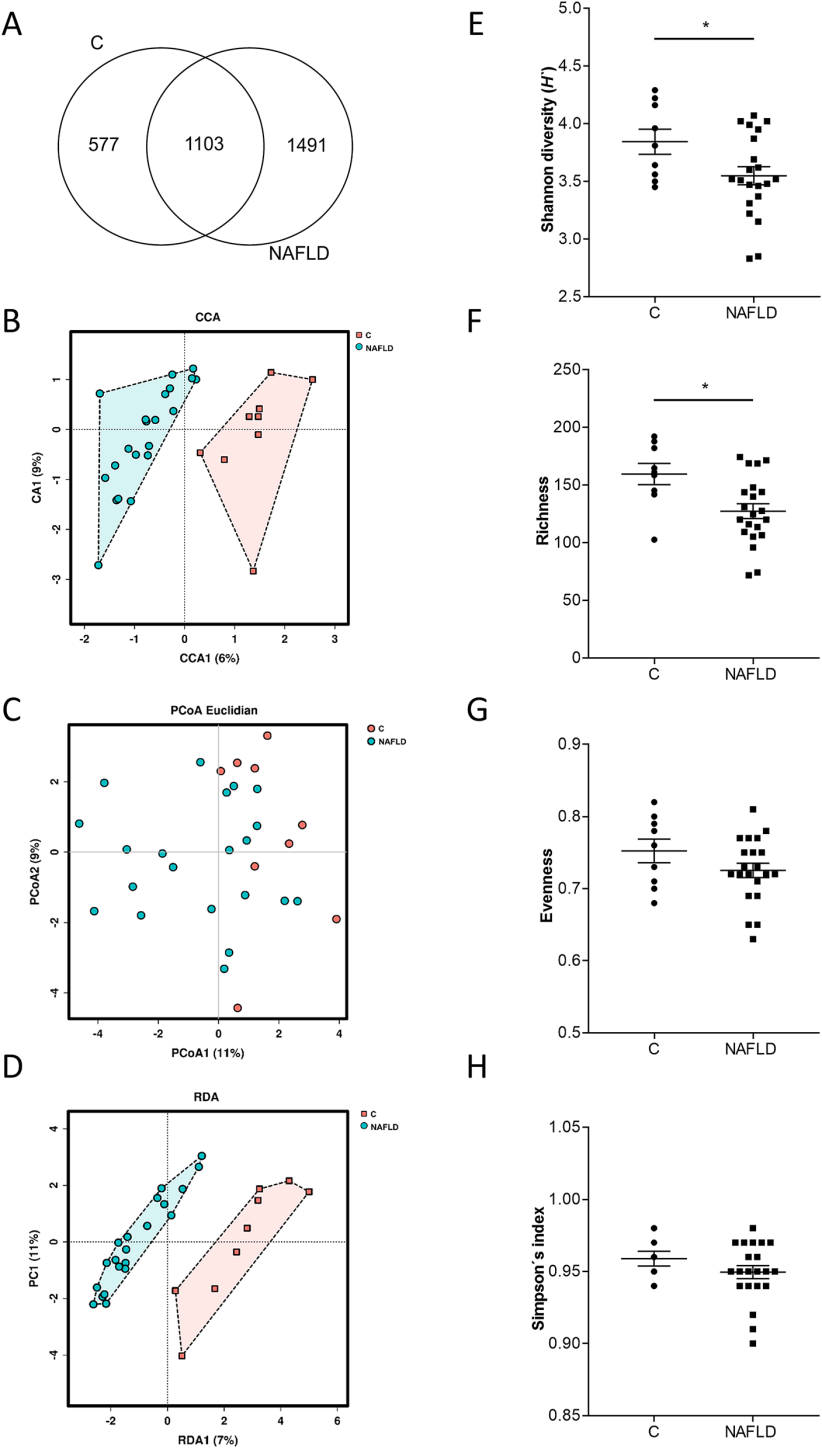
Global bacterial community structure and diversity indexes of intestinal microbiota in NAFLD patients and controls. (**A**) Venn diagram representing the operational taxonomic units (OTUs)/species of the unfiltered reads, (**B**) Principal Coordinates Analysis (PCoA) with Elucidian dissimilarity distance, (**C**) canonical correspondance analysis (CCA) and (**D**) redundance analysis (RDA), (**E**) Shannon diversity index, (**F**) species richness, (**G**) species evenness and (**H**) Simpon’s index. Each dot represents the microbial community of each proband. Data are shown as means ± SEM, n = 9 controls, n = 21 NAFLD patients, *p ≤ 0.05. *C* healthy controls, *NAFLD* patients with non-alcoholic fatty liver disease.

**Figure 2:**
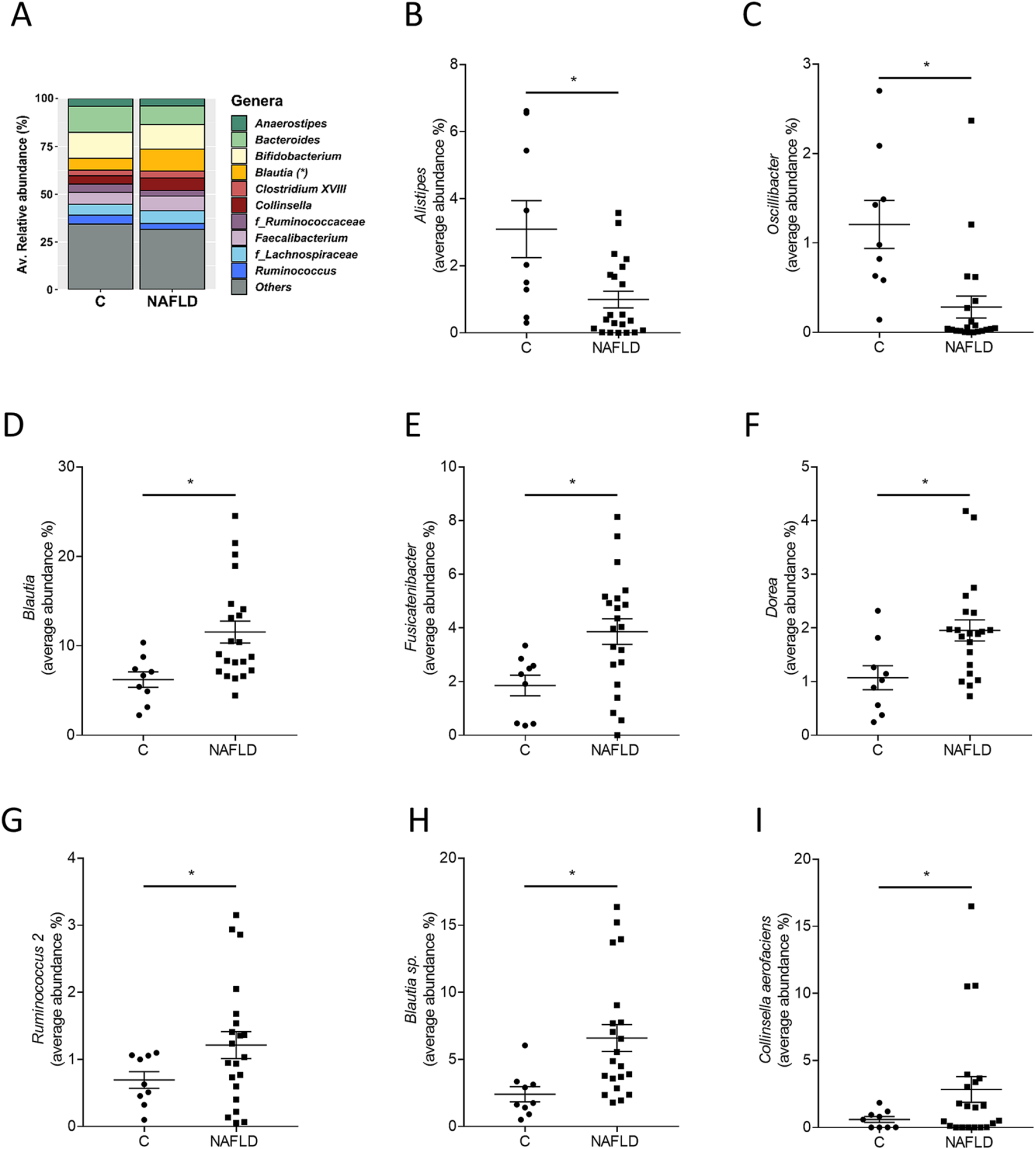
Microbial composition in feces of NAFLD patients and controls. (**A**) Relative abundance of bacterial genera in feces of NAFLD patients and controls as well as the average abundances of (**B**–**G**) genera and (**H**, **I**) species that are statistically different. Data are shown as means ± SEM, n = 9 controls, n = 21 NAFLD patients, *p ≤ 0.05. *C* healthy controls, *NAFLD* patients with non-alcoholic fatty liver disease.

While a total of 1.103 operational taxonomic units (OTUs) were shared between the NAFLD patients and controls, the number of unshared OTUs in the NAFLD patients was almost three times higher than the unshared OTUs in controls (Fig. 1A). However, most of the unshared OTUs were present in low abundances. The Principal Coordinates Analysis (PCoA) showed a clustering of microbial communities in NAFLD patients and the controls (Fig. 1B). The microbial community of both groups was statistically different when analysed by PERMONOVA (*p* = 0.009). Canonical correspondence analysis (CCA) and redundance analysis (RDA) tests indicated that NAFLD patients and controls have a unique composition (*p* = 0.001 and *p* = 0.003, respectively; Fig. 1C,D). The average similarity among replicates within the two groups was 37% in NAFLD patients and 36% in controls. The diversity of the bacterial communities measured by the Shannon’s index and species richness were statistically lower in NAFLD patients compared with controls (Fig. 1E,F). Species evenness and the Simpson’s index did not show statistical differences between the groups (Fig. 1G,H). The relative abundance of bacterial genera in feces of NAFLD patients and controls is shown in Fig. 2A. Relative abundances of *Alistipes* and *Oscillibacter* were significantly lower in feces obtained from patients with NAFLD than in controls (Fig. 2B,C). Relative abundances of *Blautia*, *Fusicatenibacter*, *Dorea* and *Ruminococcus 2* were significantly higher in feces obtained from patients with NAFLD than in feces of controls (Fig. 2D–G, *p* ≤ 0.05 for all). The result of the Linear discriminant analysis coupled with Effect Size (LEfSe) showed that the genera *Blautia*, *Fusicatenibacter*, *Dorea*, *Clostridium XIVa* and an unclassified member of *Succinivibrionaceae* (Linear Discriminant Analysis score, LDA > 2.5) were more abundant in NAFLD patients than in controls whereas nine other taxa were predominately found in controls (Figure S1). At species level, *Blautia* sp. and *Collinsella aerofaciens* were detected in higher abundance in feces of NAFLD patients in comparison to controls (Fig. 2H,I).

### Markers of intestinal permeability in NAFLD patients and controls in relation to *TLR1* mRNA expression in PBMCs

**Figure 3:**
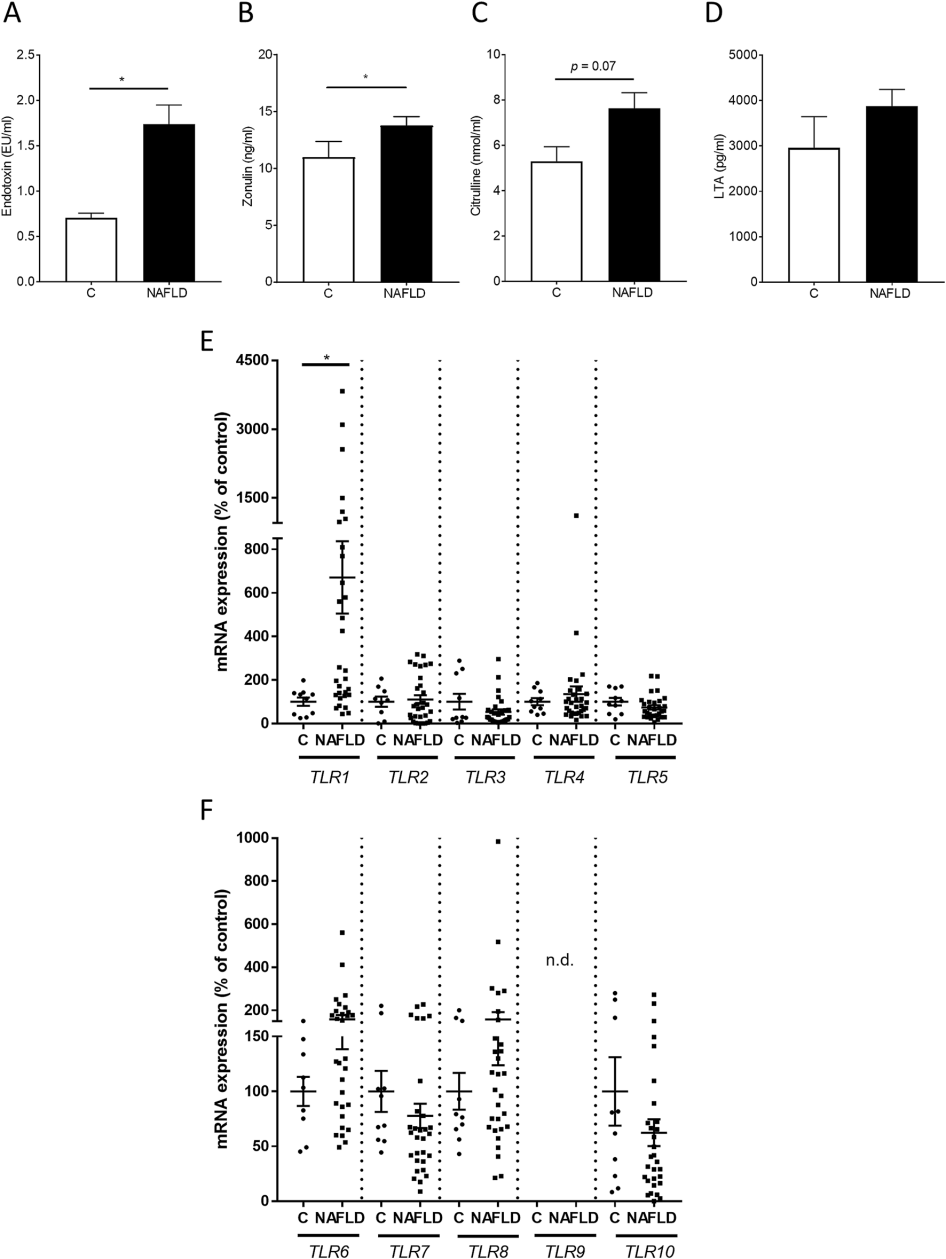
Markers of intestinal permeability and TLR expression in human PBMCs of NAFLD patients and controls. (**A**) Endotoxin, (**B**) zonulin, (**C**) citrulline and (**D**) lipoteichoic acid in plasma as well as (**E**) *TLR1* to *TLR5* and (**F**) *TLR6* to *TLR10* mRNA expression. Data are shown as means ± SEM, n = 10–14 controls, n = 31–36 NAFLD patients, *p ≤ 0.05. *C* healthy controls, *LTA* lipoteichoic acid, *NAFLD* patients with non-alcoholic fatty liver disease, *PBMCs* peripheral blood mononuclear cells, *TLR* toll-like receptor.

Bacterial endotoxin levels in plasma and zonulin concentration in serum were significantly higher in patients with NAFLD than in controls (Fig. 3A,B). Citrulline concentrations trended to be numerically higher in NAFLD patients compared to controls (*p* = 0.07; Fig. 3C). In contrast, plasma levels of lipoteichoic acid (LTA), being a ligand of TLR2 and 6 as well as TLR1^15^, were not different between controls and NAFLD patients (Fig. 3D).

### Expression of TLRs and dependent signaling cascades in NAFLD patients and controls

Apart from *TLR9* being under the level of detection, expression of *TLR1-10* was detectable in human PBMCs isolated from both, patients with NAFLD and controls (Fig. 3E,F). However, only expression of *TLR1* mRNA differed significantly between groups with *TLR1* expression being significantly higher in PBMCs isolated from patients with NAFLD than in controls. Expression of the TLR adaptor protein *MYD88* as well as expression of TIR domain containing adaptor protein (*TIRAP*) and interferon regulatory factor 3 (*IRF3*) were similar between groups (Table S1).

### Correlation analysis of *TLR1* expression in PBMCs with intestinal microbiota and markers of intestinal permeability

**Figure 4:**
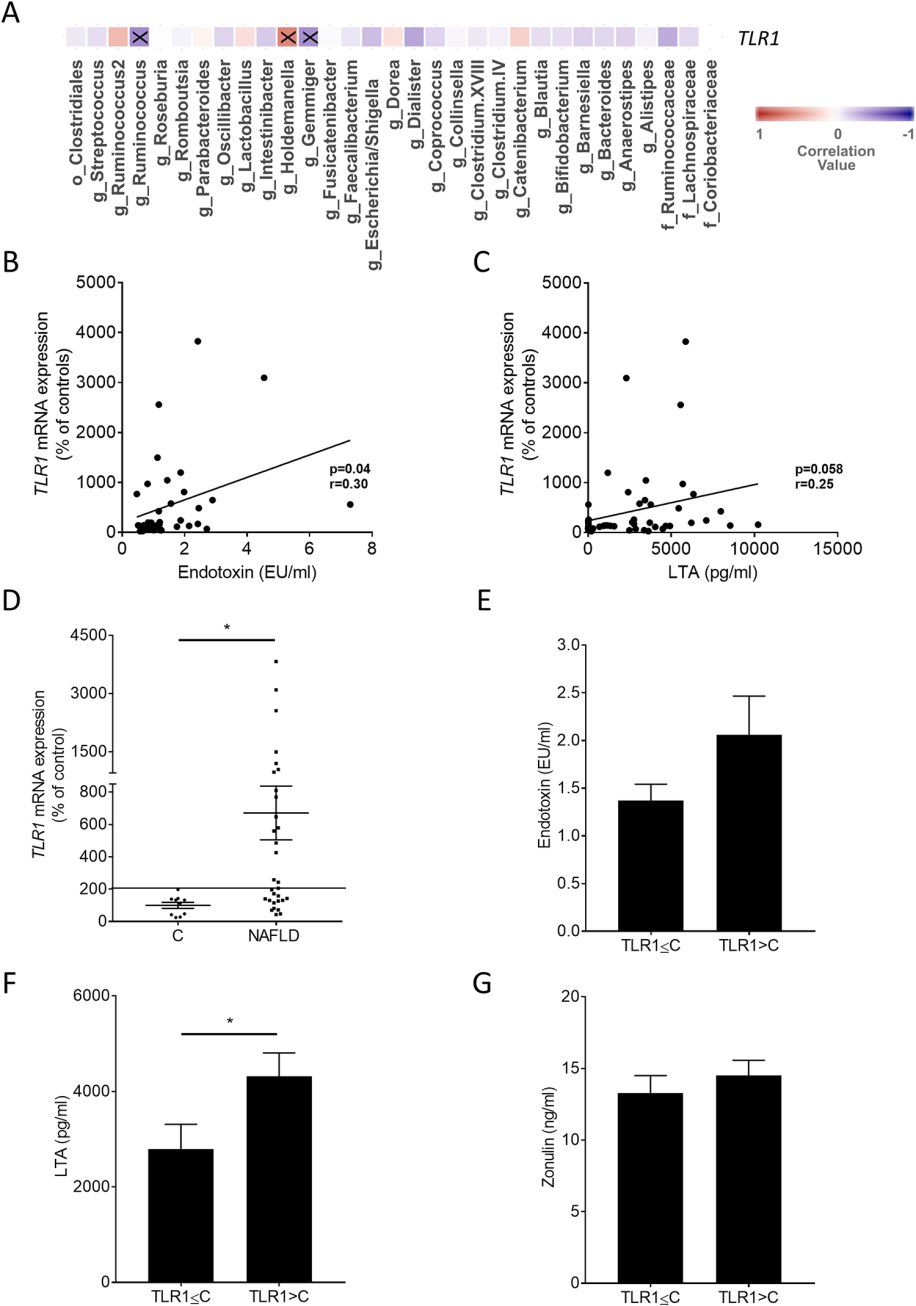
Correlation analyses of *TLR1* mRNA expression and bacterial taxa or markers of intestinal permeability as well as markers of intestinal permeability stratified to *TLR1*. (**A**) Heatmap showing the Pearson correlation between the bacterial taxa and *TLR1* and correlation analysis of *TLR1* mRNA expression in human PBMCs with (**B**) endotoxin and (**C**) LTA concentrations in plasma. (**D**) *TLR1* mRNA expression, concentration of (**E**) endotoxin, (**F**) LTA and (**G**) zonulin stratified according to *TLR1.* Letters before the name of bacterial communities represent the taxonomical level, g = genus, f = family, o = order. Higher values in red above 0 show a positive correlation, while values lower than 0 in blue indicate a negative correlation. Correlations marked with an X are statistically significant. Line indicates the cut-off of 200% *TLR1* mRNA expression. *TLR1* expression ≤ C, patients with NAFLD showing a *TLR1* mRNA expression in PBMCs ≤ *TLR1* mRNA expression in PBMCs of C; *TLR1* expression > C, patients with NAFLD showing a *TLR*1 mRNA expression in PBMCs > *TLR1* mRNA expression in PBMCs of C. Data are shown as means ± SEM, n = 7–14 controls, n = 16–31 NAFLD patients, *p ≤ 0.05. *C* healthy controls, *LTA* lipoteichoic acid, *NAFLD* patients with non-alcoholic fatty liver disease, *PBMCs* peripheral blood mononuclear cells, *TLR* toll-like receptor.

To determine if relative abundance of certain bacterial genera or strains detected in feces of patients with NAFLD and controls as well as markers of intestinal permeability were related to *TLR1* mRNA expression in PBMCs, a correlation analysis was performed. The genus *Holdemanella* was significantly positive related to the mRNA expression of *TLR1* in PBMCs. In contrast, for the genera *Ruminococcus* and *Gemmiger*, a significantly negative correlation with mRNA expression of *TLR1* in PBMCs was found (Fig. 4A; Table S2). Levels of endotoxin in plasma were significantly positive correlated with *TLR1* mRNA expression in PBMCs with a similar trend being also found for LTA plasma levels and *TLR1* mRNA expression in PBMCs (Fig. 4B,C). However, no associations between further markers of intestinal integrity and the mRNA expression of *TLR1* in PBMCs were found (data not shown).

**Figure 5:**
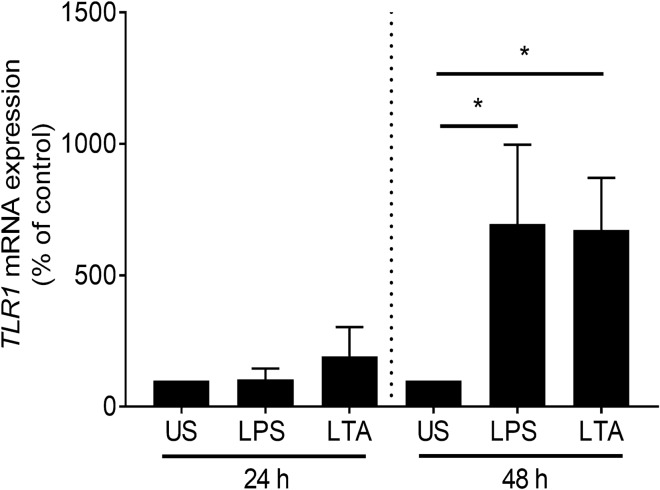
*TLR1* mRNA expression in human PBMCs in healthy probands. Human PBMCs were stimulated ex vivo with 100 ng/ml LPS or 10 µg/ml LTA for 24 or 48 h. Data are shown as means ± SEM, n = 6, *p ≤ 0.05. *LPS* lipopolysaccharide, *LTA* lipoteichoic acid, *PBMCs* peripheral blood mononuclear cells, *TLR* toll-like receptor, *US* unstimulated.

When further analyzing mRNA expression of *TLR1* in PBMCs of patients with NAFLD, it was found that *TLR1* mRNA was clustered with 14 NAFLD patients showing *TLR1* mRNA expression in PBMCs similar or below to that of controls and 17 NAFLD patients having *TLR1* mRNA expression in PBMCs above that of controls (highest control: 198%, Fig. 4D). Interestingly, when clustering plasma endotoxin, LTA and zonulin levels accordingly, LTA plasma levels of NAFLD patients with a high *TLR1* mRNA expression (> 198%) were found to be significantly higher than those of patients with low *TLR1* mRNA expression in PBMCs (Fig. 4F). Differences alike were not found for endotoxin and zonulin levels (Fig. 4E,G). To determine if endotoxin and LTA, respectively, affect *TLR1* expression in human PBMCs, PBMCs isolated from healthy young adults were challenged with 100 ng/ml lipopolysaccharide (LPS) or 10 µg/ml LTA for 24 and 48 h and expression of *TLR1* was determined. While after 24 h, expression of *TLR1* was unchanged in cells challenged with LPS or LTA, *TLR1* mRNA expression was super-induced after 48 h (Fig. 5).

### Expression of *Tlrs* in PBMCs and of hepatic *Tlr1* in mice with diet-induced NAFLD as well as plasma levels of LTA and endotoxin in portal vein

**Figure 6:**
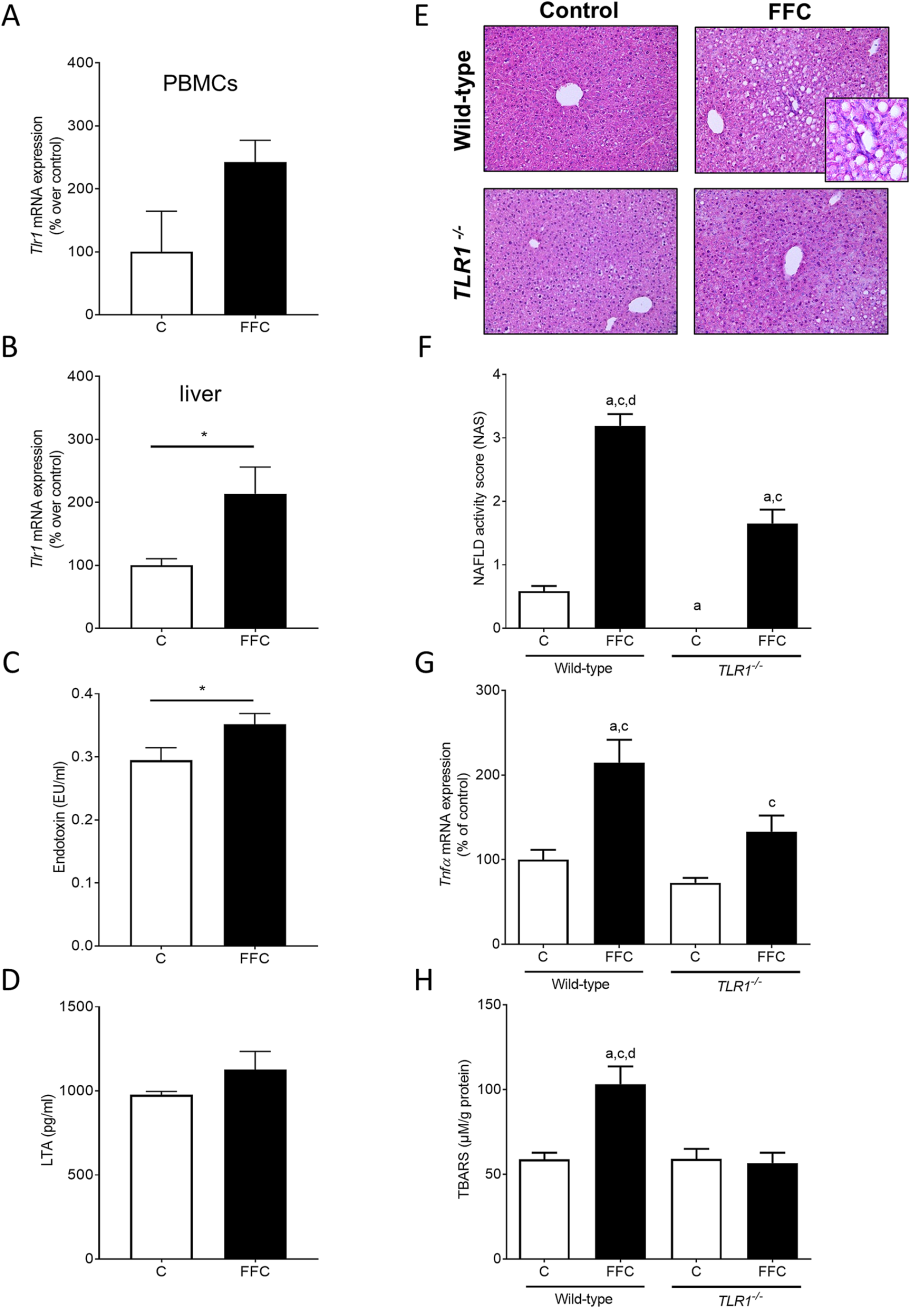
Effect of TLR1 on markers of intestinal permeability and indices of liver damage in FFC-fed mice. *Tlr1* mRNA expression in (**A**) PBMCs and (**B**) liver tissue, (**C**) endotoxin concentration as well as (**D**) LTA levels in plasma of C- and FFC-fed wild-type mice. (**E**) Representative pictures (magnification ×200 and ×400) of hematoxylin and eosin staining and (**F**) NAFLD activity score (NAS) in liver tissue, (**G**) mRNA expression of *Tnfα* in liver tissue and (**H**) concentration of TBARS in liver tissue of C- and FFC-fed *TLR1*^−/−^ and wild-type mice. Data are shown as means ± SEM, n = 5–8, except of (**A**) as yield of PBMCs was rather low, 2 samples were pooled respectively and for (**D**): n = 4 in C-fed mice, *p ≤ 0.05 between C- and FFC-fed wild-type mice; ^a^*p* ≤ 0.05 compared to C-fed wild-type mice; ^c^*p* ≤ 0.05 compared to C-fed *TLR1*^−/−^ mice; ^d^*p* ≤ 0.05 compared to FFC-fed *TLR1*^−/−^ mice. *C* control diet, *FFC* fat-, fructose- and cholesterol-rich diet, *LTA* lipoteichoic acid, *PBMCs* peripheral blood mononuclear cells, *TBARS* thiobarbituric acid reactive substances, *Tlr* toll-like receptor, *Tnf* tumor necrosis factor.

To determine if changes in *Tlr1* mRNA expression in PBMCs are related to changes in expression of *Tlr1* in liver, mice were either pair-fed a fat-, fructose- and cholesterol-rich diet (FFC) for six weeks to induce NAFLD or a control diet (C). As the yield of PBMCs and mRNA was rather low, samples of two mice were pooled so that a total of four samples obtained from eight mice per group were analyzed. Accordingly, no statistical analysis was performed. In line with the findings in humans, *Tlr1* expression was also markedly higher in PBMCs isolated from mice with diet-induced NAFLD than in C-fed mice (Fig. 6A). Furthermore, *Tlr1* mRNA expression in liver tissue was also significantly higher in mice with NAFLD than in controls (Fig. 6B). Also, in line with the findings in humans, concentrations of bacterial endotoxin in plasma obtained from the portal vein of mice with NAFLD were also significantly higher than in control animals while LTA levels, varying considerably between animals, did not differ (Fig. 6C,D).

### Liver histology, clinical parameters and markers of inflammation as well as lipid peroxidation in wild-type mice and ***TLR1***^−/−^ mice fed a diet to induce NAFLD

To determine if TLR1 is critical in the development of NAFLD, *TLR1*^−/−^ mice and wild-type mice were fed a FFC diet for eight weeks. Liver weight and liver to body weight ratio were significantly higher in FFC-fed *TLR1*^−/−^ and wild-type mice than in C-fed groups (Table 2). Development of steatosis and even more of early signs of inflammation were markedly attenuated in FFC-fed *TLR1*^−/−^ when compared to FFC-fed wild-type animals. Indeed, NAFLD activity score (NAS) was significantly lower in *TLR1*^−/−^ mice fed the FFC diet when compared to FFC-fed wild-type mice (Fig. 6E,F). However, NAS in FFC-fed *TLR1*^−/−^ mice was still significantly higher than in both control groups. Expressions of *F4/80* and tumor necrosis factor alpha (*Tnfα*) mRNA were also significantly higher in livers of FFC-fed wild-type mice when compared to both control groups while in *TLR1*^−/−^ mice fed the FFC, expression was only significantly higher than in C-fed *TLR1*^−/−^ mice (Table 2; Fig. 6G). In line with these findings, concentration of thiobarbituric acid reactive substances (TBARS) in liver tissue was only significantly higher in FFC-fed wild-type mice than in both C-fed mice. Concentration of TBARS in livers of *TLR1*^−/−^ mice fed the FFC was at the level of controls (Fig. 6H). Neither alanine aminotransferase (ALT) nor aspartate aminotransferase (AST) or fasting glucose levels differed between groups irrespective of diet fed (Table 2).

**Table 2:** Effect of deletion of TLR1 on caloric intake, body- and liver weight and liver damage in FFC-fed mice.

	Wild-type		*TLR1*^−/−^	
	C	FFC	C	FFC
Caloric intake (kcal/g bw)	0.45 ± 0.0	0.44 ± 0.0	0.42 ± 0.0	0.42 ± 0.0
Body weight (g)	22.1 ± 0.4	22.7 ± 0.6	22.5 ± 0.8	23.7 ± 0.6
Liver weight (g)	1.2 ± 0.1	1.6 ± 0.1^a,c^	1.1 ± 0.1	1.5 ± 0.1^a,c^
Liver:body weight ratio (%)	5.3 ± 0.2	6.9 ± 0.2^a,c^	4.9 ± 0.2	6.5 ± 0.2^a,c^
ALT (U/l)	15.7 ± 2.6	16.1 ± 1.2	16.5 ± 3.1	20.2 ± 1.5
AST (U/l)	40.8 ± 1.2	43.7 ± 2.4	44.6 ± 3.0	47.9 ± 0.8
Fasted glucose concentration (mg/dl)	102.0 ± 3.6	92.0 ± 6.3	81.0 ± 4.0	87.8 ± 7.9
*F4/80* mRNA expression (% of control)	100 ± 6.0	192.1 ± 18.5^a,c^	112.1 ± 77.8	158.4 ± 17.8^a^

Data are shown as means ± SEM, n = 5–6, ^a^*p* ≤ 0.05 compared to C-fed wild-type mice, ^c^*p* ≤ 0.05 compared to C-fed *TLR1*^−/−^ mice.

*ALT* alanine aminotransferase, *AST* aspartate aminotransferase, *C* control diet, *FFC* fat-, fructose-, and cholesterol-rich diet, *TLR* toll-like receptor.

## Discussion

NAFLD is by now the leading liver disease in the world^16^. And while results of epidemiological studies suggest that overweight and insulin resistance are the key risk factors for the development of NAFLD, results of clinical and experimental studies also suggest that changes of intestinal microbiota composition and intestinal barrier function may contribute to disease onset and progression (for overview see^17^). In line with the findings of others^18^, results of the present study suggest that NAFLD is associated with marked changes in intestinal microbiota composition and lower diversity of microbial communities. Indeed, the lower relative abundance of *Alistipes* and *Oscillibacter* species in NAFLD patients in comparison to controls is in line with the findings of others showing a reduction of *Alistipes* in patients with liver fibrosis and cirrhosis and of *Oscillibacter* in NAFLD patients^19–21^ (and for overview see^22^). Furthermore, a higher relative abundance of *Blautia*, *Fusicatenibacter* and *Dorea* as well as *Ruminococcus 2* in NAFLD patients in comparison to healthy controls was reported by others studying humans with this liver disease^19,23^. However, others reported that NAFLD was associated with a decrease in the relative abundance of *Blautia* and *Ruminococcus*^24^. Further studies are needed to determine the physiological impact of these alterations, and herein, especially the impact of changes in the abundance of specific bacteria on intestinal barrier function but also the liver.

The changes found in intestinal microbiota abundance and composition were associated with higher levels of zonulin protein, being proposed to be indicative of intestinal barrier function and integrity^25^. In line with these findings, bacterial endotoxin levels in plasma of NAFLD patients were also significantly higher, all suggesting that intestinal barrier function of NAFLD patients was impaired, and subsequently, translocation of bacterial toxins was increased. These findings are in line with the findings of others and earlier studies of our own group in adults and children with early and progressed signs of the disease all suggesting that the development and progression of NAFLD is associated with changes in intestinal barrier function and an increased translocation of bacterial endotoxin^26–29^. Somewhat contrasting the findings for bacterial endotoxin and the other markers of intestinal barrier function, LTA levels in plasma were not markedly higher in NAFLD patients; however, we found that data varied considerably and were sort of clustered. Indeed, when delineating LTA levels according to *TLR1* expression in PBMCs, we found that those patients with a *TLR1* expression higher than the highest expression found in healthy controls also had significantly higher LTA levels in their blood than those with an expression at the level of controls. In line with these findings, in mice with a FFC-induced NAFLD, *Tlr1* mRNA expression in PBMCs was also markedly higher going also along with significantly higher bacterial endotoxin levels while LTA levels, varying considerably, were similar to controls. It might be that in mice, duration of feeding was not yet sufficient to stably increase LTA levels in plasma, yet. Furthermore, it has been shown before that similar to TLR4, surface resident TLR1 forming heterodimers with TLR2 or TLR6, clears its ligands through forming endosomes^30^. It could very well be, that in FFC-fed mice displaying only early signs of steatosis with beginning inflammatory alterations, clearance of LTA through TLR1 in intestinal tissue and liver was efficient enough to avoid a marked increase of LTA in the portal vein. Indeed, *Tlr1* mRNA expression was significantly higher in livers of FFC-fed mice when compared to control animals. Interestingly, in human samples, similar relations were not found for TLR2 or TLR6, both shown to form heterodimers with TLR1 and to be critical for the responsiveness of TLR1 to bacterial toxins^31^. However, while expressions of *TLR2* and *TLR6* were not elevated in PBMCs of patients with NAFLD, expression of both receptors was still detectable. Further studies are needed to determine the interplay of TLR1, TLR2 and TLR6 in PBMCs in NAFLD patients. Somewhat supporting the hypothesis that *TLR1* expression is directly related to plasma levels of bacterial toxins, and herein, especially LTA and endotoxin, expression of *TLR1* mRNA was found to be significantly induced upon stimulation of both, LTA and LPS. Furthermore, expression of *TLR1* in PBMCs of NAFLD patients and controls was positively related to plasma levels of bacterial endotoxin. It has been shown by others before, that number of TLR1^+^ monocytes is increased upon stimulation with LTA^32^. Furthermore, it has been shown that TLR1 is expressed in all leukocytes^33^ and that in the present of TLR2 may also be activated by certain LPS preparations^31^.

Interestingly and contrasting the findings of us and others assessing expression of TLRs in liver tissue of both rodents and humans with NAFLD^9,10^ but also studies in PBMCs isolated from patients with chronic hepatitis B and chronic hepatitis B-related liver failure^34^, only *TLR1* expression was found to be higher in PBMCs of NAFLD patients when compared to healthy controls. Differences between the studies of Wang et al. and our study could have resulted from differences in disease etiology, e.g. virus infection vs. NAFLD. It could also be that the difference between the findings in liver and blood cells are related to the fact that PBMCs are a mixed immune cell population^35^. Indeed, it has been shown before that while TLRs are traditionally associated with cells of the innate immune response, cells of adaptive immunity like the different classes of T and B lymphocytes have been shown to express a variety of TLRs^33^; however, at substantially lower magnitude than innate immune cells^36^. Furthermore, when interpreting these data, it also needs to be kept in mind that in a recently published study, it was shown that composition of PBMCs obtained from NAFLD patients differs from that of controls^37^ with lower frequencies being found for CD3^+^ and CD8^+^ T cells, CD56^dim^NK cells and MAIT cells but elevated frequencies of CD4^+^ T cells and Th2 cells. To what extend these differences added to the differences or lack of induction of TLRs in NALFD patients found in the present study needs to be clarified in future studies.

Expression of *TLR1* in PBMCs of NAFLD patients and healthy controls was related to a higher relative abundance of the gram-positive bacterial genus *Holdemanella* and a lower relative abundance of *Gemmiger* and *Ruminococcus* in stool. Results of in vitro studies suggest that certain bacterial strains can alter expression of several TLRs in epithelial cell lines^38^; however, effects on the expression of *TLR1* mRNA were limited in these studies. Studies assessing the effects of changes of bacterial composition on *TLR* expression in PMBCs with respect to certain bacteria to our knowledge are so far lacking. Also, if and how *Holdemanella*, *Gemmiger* or *Ruminococcus* might have added to the induction of *TLR1* mRNA expression in PBMCs in the present study have to be determined in future studies.

Taken together, results of the present study suggest that intestinal microbiota, and herein, apperently specific genus like *Holdemanella* and bacterial toxins are positively related to *TLR1* expression in PBMCs of NAFLD patients and that expression of *TLR1* can be induced by both LPS and LTA. However, results by no means preclude that other TLRs in PBMCs may also be altered in patients with NAFLD as in the present study, only patients with fatty liver or NASH were studied. Rather, our results suggest that in patients with NAFLD, certain TLRs, and herein, probably especially TLR1 are induced in PBMCs and that this might also reflect elevated levels of certain bacterial toxins in peripheral blood.

It has been shown before by us and others that a genetic deletion or specific inhibition of certain TLRs, e.g. TLR4 and TLR9, may dampen or even abolish the development of NAFLD^39–41^ while for others like TLR2 was even found that a deletion may even exacerbate the development of NAFLD in mice^42^. In the present study, we found that the genetic deletion of TLR1 in mice fed a FFC is associated with a marked protection against the development of NAFLD. Indeed, results suggest that in livers of *TLR1*^−/−^ mice with beginning inflammatory alterations were dampened. It has been suggested before, that TLR1-dependent signaling cascades are involved in mediating inflammatory response to various bacteria derived stimuli. Whether the protective effects of the loss of TLR1 in FFC-fed mice found in the present study are related to a lower sensitivity to LTA or LPS or both remains to be determined in future studies. Also, as we used whole body knockout mice and the yield of PBMCs of FFC-fed mice was too low to perform a statistical analysis, the results obtained in mice can only in part be related to the findings in humans and the question if an induction of TLR1 in PBMCs in NAFLD patients impacts the development of the disease will have to be addressed in future studies. Still, together with the results of others, results of the present study add further support to the role of bacterial toxins and TLR signaling in the development of NAFLD^10,43^.

The present study has some limitations. Specifically, the number of stool samples for analysis of the microbial communities in the control group is rather low (controls: n = 9). This has to be taken into consideration when interpreting the results of the present study. Despite this shortcoming, our findings that diversity of intestinal microbiota of healthy normal weight subjects differs from that of NAFLD patients is in line with those of others^44^. Although, while not reaching the level of significance (Fisher’s exact test *p* = 0.0635), gender differences might have impacted the results of the present study. Indeed, gender has been shown to affect the development and progression of NAFLD, with women have a lower risk to develop NAFLD but a higher risk in progression of the disease than men^45^. In addition, as the yield of isolated PBMCs and resulting RNA of FFC-fed mice and their respective controls was too low, it was not possible to perform a proper statistical analysis of the data. Accordingly, the question if *Tlr1* mRNA expression in PBMCs is also altered in FFC-fed mice suffering from NAFLD remains to be determined.

Taken together, results of the present study further bolster the hypothesis that changes in intestinal microbiota composition in patients with NAFLD are associated with alterations of intestinal barrier function as well as increased bacterial endotoxin levels. Our results also suggest that contrary to the findings in liver tissue, in peripheral blood cells obtained from patients with NAFLD, only expression of *TLR1* mRNA is increased and that this may depend upon LTA blood levels. Further studies are needed to determine clinical implications of these alterations, especially, in regard to disease progression. Furthermore, results of the present study also suggest that TLR1 may also be a critical factor in the development of NAFLD and that a genetic deletion of this receptor dampens the development of NAFLD; however, it remains to be determined, if these beneficial effects depend upon a decreased sensitivity towards LTA or other bacterial toxins and if effects alike are also found in humans.

## Methods

### Study subjects and design

A total of 37 patients with known NAFLD as diagnosed by ultrasound according to current guidelines and with no relevant alcohol intake (defined as < 20 g/d for women and < 30 g/d for men) as well as 15 age-matched normal weight disease-free controls were recruited at the outpatient hepatology clinic of the University Medical Centre of the Johannes Gutenberg-University, Mainz, Germany and the Department of Nutritional Sciences, Vienna, Austria. The `Ethikkommission der Landesaerztekammer Rheinland-Pfalz´ (Germany) and the ethics committee of the Medical University Vienna (Austria) approved the studies in accordance with the Helsinki II Declaration and all patients and controls enrolled gave their written informed consent to participate in the study. NAFLD patients were part of the prospectively enrolling NUCES-NASH study (NCT02366052; Nutritional Counseling vs. Nutritional Supplements for NASH—a Randomized Prospective, Open Label Pilot Study) and the ENDO-META study (NCT03482284). None of the patients or controls followed any dietary restrictions or took antibiotics four weeks prior or at sample collection. Patients with NAFLD were included in the NUCES-NASH study when they had elevated M30 antigen levels (cut-off: > 200 to 800 U/L) and were diagnosed with hepatic steatosis by ultrasound or histologically confirmed NASH. The degree of hepatic steatosis was classified into mild (grade 1), moderate (grade 2) or severe (grade 3) according to a semi-quantitative scoring system^46^ in a standardized manner by a trained hepatologist. The term NAFLD was used to describe the NAFLD patients throughout the manuscript as liver histology was not accessible in all patients. Data presented were obtained at the screening visit of fasted patients and controls.

### Animal experiments

For all animal experiments, female 8–10 weeks old *TLR1*^−/−^ and C57BL/6 mice (Jackson Laboratory, Bar Harbor, ME, USA and Janvier SAS, Le Genest-Saint-Isle, France) were housed in a specific-pathogen-free barrier facility accredited by the Association for Assessment and Accreditation of Laboratory Animal Care. All experiments were carried out under controlled conditions with mice having free access to tap water at all times. All procedures were approved by the local institutional animal care and use committee (`Regierungspraesidium Stuttgart´ and `Federal Ministry Republic of Austria Education, Science and Research´) and animals were handled in accordance to the European Convention for the Protection of Vertebrate Animals used for Experimental and Other Scientific Purposes and carried out in compliance with the ARRIVE guidelines. Mice were adapted to a liquid control diet (C; 69E% carbohydrates, 12E% fat, 19E% protein; Ssniff, Soest, Germany) and then randomly assigned to the different feeding groups. For the isolation of PBMCs, wild-type mice (n = 8) were pair-fed the C diet or a fat-, fructose- and cholesterol-rich diet (FFC; 60E% carbohydrates, 25E% fat, 15E% protein with 50% wt/wt fructose and 0.16% wt/wt cholesterol; Ssniff, Soest, Germany) for six weeks after the adaption to the liquid diet. To determine the role of TLR1 in the development of NAFLD, *TLR1*^−/−^ (n = 5–6/group) and wild-type mice (n = 6/group) were either pair-fed the FFC or the C diet for eight weeks*.* As problems were encountered with breeding knockout mice and one mouse was lost when collecting fasting blood samples, it was only possible to analyze samples of n = 5–6 *TLR1*^−/−^ mice. For pair-feeding, caloric intake was adjusted daily to the group with the lowest caloric intake. Body weight was assessed weekly. After six weeks of feeding, *TLR1*^−/−^ and wild-type mice were fasted for 6 h and blood was taken from retrobulbar venous plexus under isoflurane anesthesia. Fasting blood glucose levels were assessed with a glucometer (Bayer Consumer Care AG, Basel, Switzerland).

### Clinical measures, non-invasive scores of advanced hepatic fibrosis and vibration controlled transient elastography (VCTE)

Fasting blood samples were obtained from all NAFLD patients and controls to assess ALT, AST and γ-GT activity as well as triglycerides, total cholesterol, LDL and HDL cholesterol, CRP, glucose, insulin and uric acid concentration (Routine lab of the University Medical Centre of the Johannes Gutenberg-University, Mainz, Germany and IhrLabor, Vienna, Austria). In addition, concentrations of leptin (Hölzel Diagnostika Handels GmbH, Cologne, Germany), adiponectin (TECOmedical AG, Sissach, Switzerland) and PAI-1 (LOXO GmbH, Dossenheim, Germany) were measured with commercially available ELISA kits according to the manufacturers’ instructions. Type 2 diabetes, hypertension, hyperlipidemia and the metabolic syndrome were defined according to the definitions of the Joint Scientific Statement for Harmonizing the Metabolic Syndrome^47^. In NAFLD patients, NAFLD fibrosis score with age-adjusted cut-offs, APRI and Fib-4 score were calculated to predict advanced fibrosis as published^48^. Furthermore, VCTE was performed using Fibroscan 402 (Echosens, Paris, France) as part of the standard liver work-up by trained study coordinators using a standardized protocol in NAFLD patients. According to published findings, a cut-off of > 12.0 kPa was used to predict advanced fibrosis^49,50^.

### Isolation of PBMCs

PBMCs were isolated from whole blood specimen of humans and mice as described in detail previously^51^. In brief, whole blood was mixed with PBS and gently layered onto histopaque-1077 (Sigma-Aldrich Handels GmbH, Vienna, Austria). After density gradient centrifugation, PBMCs were collected from the plasma/histopaque interface. PBMCs were either frozen or lysed with Trizol (peqGOLD, Trifast, Peqlab, Germany) until further analyses or subsequent RNA isolation.

### Stimulation of PBMCs ex vivo

Human PBMCs were stimulated with 100 ng/ml LPS (serotype O55:B5; Sigma-Aldrich, Steinheim, Germany) or 10 µg/ml LTA (Sigma-Aldrich, Steinheim, Germany) for 24 and 48 h, respectively. Cells were lysed in Trizol (peqGOLD TriFast, peqlab, Erlangen, Germany) and RNA was isolated.

### Markers of intestinal permeability

Bacterial endotoxin and LTA levels in mouse and human plasma were assessed using commercially available assays (Charles River, Germany and Novatein Biosciences, MA, USA). Zonulin and citrulline blood concentrations were assessed using commercially ELISAs (Immundiagnostik AG, Bensheim, Germany and Hölzel Diagnostika Handels GmbH, Cologne, Germany) following the instructions of the manufacturer.

### Scoring of liver histology and clinical markers in mice

Mouse liver sections (4 µm) of paraffin-embedded tissue were stained with hematoxylin and eosin to evaluate liver histology using NAS according to Kleiner et al.^52^. Activities of ALT and AST were determined in plasma in the routine laboratory of the University Hospital of Jena, Germany (Architect, Abbott, Wiesbaden, Germany).

### RNA extraction and real-time RT PCR

RNA was isolated from frozen mouse and human PBMCs and mouse liver tissue using Trizol (peqGOLD TriFast, Peqlab, Germany) and reverse transcribed using a commercially available cDNA synthesis kit (Promega, Madison, WI, USA). Primers designed using the software Primer 3 are shown in Table S3. Real-time PCR was carried out and calculations were performed as previously detailed^51^.

### TBARS assay

To determine TBARS, formed as a byproduct of lipid peroxidation^53^, liver tissue was homogenized in RIPA buffer supplemented with protease inhibitors. After precipitation with 10% trichloroacetic acid, supernatant was incubated with thiobarbituric acid for 10 min at 95 °C. Concentration of TBARS was measured at 532 nm with a photometer (SpectraMax, Molecular Devices, San Jose, CA, USA).

### Illumina amplicon sequencing of fecal microbiota

Fecal samples from NAFLD patients (n = 21) and controls (n = 9) were analysed. All other patients and controls were either incompliant with sample collection or failed to transport samples appropriately to the laboratory. Bacterial DNA was extracted using FastDNA SPIN Kit for Soil (MP Biomedicals, Solon, OH, USA) following the manufacturer's instructions and Illumina amplicon sequencing of fecal microbiota was carried out as detailed previously^54–56^. In brief, the V1-2 region of 16S rRNA gene was amplified with a two-step PCR using Taq PrimeSTAR HS DNA (Clontech Laboratories, Mountain View, CA, USA). No negative control corrections were applied as PCR products were discarded if there was any evidence of product formation in the negative control when being checked on an agarose gel. Amplicons were purified and normalized using the SequalPrep Normalization Plate Kit (Thermo Fisher Scientific, Waltham, MA, USA). Samples were sequenced on an Illumina MiSeq platform using 250 bp paired-end sequencing chemistry. An average of 39.432 ± 5.971 raw reads per sample were quality filtered, assembled and aligned using Mothur pipeline^57^. Reads were clustered at 97% identity into 697 OTUs. Only OTUs present at an average abundance higher than 0.5% and a sequence length > 250 bp were considered for further analysis. The closest representative was manually identified using seqmatch from RDP^58^. Sequences were submitted to European Nucleotide Archive under the accession number PRJEB41058.

### Statistical analysis

All data are presented as means ± standard error of mean (SEM). Bartlett’s test was applied to test homogeneity of variances and data were log-transformed if unequal before performing further statistical tests. For analysis age and gender distribution between NAFLD patients and controls, a Fisher’s exact test was performed. For comparison of data of NAFLD patients and controls as well as of C- and FFC-fed mice an unpaired t-test was used while a two-factorial analysis of variance (ANOVA) was used to identify statistical differences between wild-type and *TLR1*^−/−^ mice fed C or FFC followed by Tukey’s post hoc test. *P* ≤ 0.05 was defined to be significant.

To analyze Illumina amplicon sequencing data PRIMER-E 7 (Plymouth Marine Laboratory, Plymouth, UK) was used as detailed before^59^. For analysis of similarity (ANOSIM), a one-way permutation-based hypothesis testing was used with differences defined as significant if *p* ≤ 0.05. Similarity percentage analysis (SIMPER) was used as detailed by Clarke and Warwick^59^ to calculate the percentage of similarity and dissimilarity and to determine which OTUs contribute to differences found in NAFLD patients and controls. Samples were standardized by total and a similarity matrix was created using Bray–Curtis coefficient^60^. Processed reads were imported to Calypso 8.84 for data analysis and visualization. The recommended program settings were used for standard filters for low sequence reads and Hellinger transformation (square root of total sum normalization). Hierarchical clustering with Euclidean distance and ordination of the community structures were visualized using PCoA and principal component analysis (PCA), CCA and RDA. To analyze the diversity indices, samples were rarefied to a read depth of 16.386 and an ANOVA was used to identify statistical differences between NAFLD patients and healthy controls. LEfSe was used to determine the bacteria most likely to explain differences between NAFLD patients and controls. Differences in the abundance of OTUs of interest between groups were evaluated using the unpaired Welch t-test.

## Supplementary Information

Supplementary Information.

## Data Availability

Illumina amplicon sequences were submitted to European Nucleotide Archive under the accession number PRJEB41058.

## Abbreviations

γ-GT: Gamma glutamyl transferase
ALT: Alanine aminotransferase
AST: Aspartate aminotransferase
BMI: Body mass index
C: Control diet or healthy controls
CCA: Canonical correspondence analysis
CRP: C-reactive protein
FFC: Fat-, fructose- and cholesterol-rich diet
IRF3: Interferon regulatory factor 3
LDA: Linear Discriminant Analysis score
LEfSe: Linear discriminant analysis coupled with Effect Size
LPS: Lipopolysaccharide
LTA: Lipoteichoic acid
MYD88: Myeloid differentiation primary response 88
NAFLD: Non-alcoholic fatty liver disease
NAS: NAFLD activity score
NASH: Non-alcoholic steatohepatitis
OTUs: Operational taxonomic units
PAMP: Pathogen-associated molecular pattern
PAI-1: Plasminogen activator inhibitor 1
PBMCs: Peripheral blood mononuclear cells
PCoA: Principal Coordinates Analysis
RDA: Redundance analysis
TBARS: Thiobarbituric acid reactive substances
TIR: Toll/interleukin-1 receptor
TIRAP: TIR domain containing adaptor protein
TLRs: Toll-like receptors
Tnfα: Tumor necrosis factor alpha
TRAM: TRIF-related adaptor molecule
TRIF: TIR-domain-containing adapter-inducing interferon β
VCTE: Vibration controlled transient elastography
