# Supplementary material for: Toll-like receptor 1 as a possible target in non-alcoholic fatty liver disease
Source: Sci Rep. 2021 Sep 8;11:17815. doi: 10.1038/s41598-021-97346-9 (PMC8426394; doi:10.1038/s41598-021-97346-9)
Supplement: Supplementary file 1 — Supplementary Information. [file 41598_2021_97346_MOESM1_ESM.pdf]

Toll-like receptor 1 as a possible target in non-alcoholic fatty liver disease

Anja Baumann[1,#], Anika Nier[1,#], Angélica Hernández-Arriaga[2], Annette Brandt[1], Maria J. Lorenzo Pisarello[1], Cheng J. Jin[3], Esther Pilar[1], Amélia Camarinha-Silva[2], Jörn M. Schattenberg[4], Ina Bergheim[1,*]

[1]Department of Nutritional Sciences, Molecular Nutritional Science, University of Vienna, Vienna, Austria

[2]Institute of Animal Science, University of Hohenheim, Stuttgart, Germany

[3]Institute of Nutrition, SD Model Systems of Molecular Nutrition, Friedrich-Schiller-University Jena, Jena, Germany

[4]Metabolic Liver Research Program, Department of Internal Medicine I, University Medical Center of the Johannes Gutenberg-University, Mainz, Germany

[#]Authors contributed equally to this work

***Corresponding author:** Ina Bergheim, Ph.D.
University of Vienna
Department of Nutritional Sciences
Molecular Nutritional Science
Althanstraße 14 (UZA2)
A-1090 Vienna
Phone: +43-1-4277-54981
E-Mail: ina.bergheim@univie.ac.at

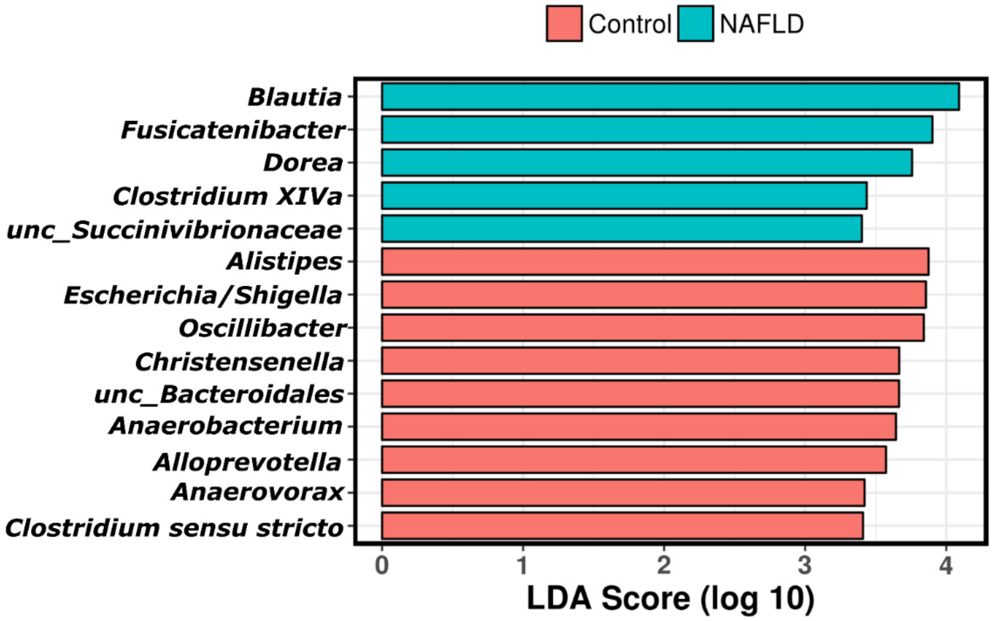

Figure S1. Histogram of the Linear Discriminant Analysis score (LDA) (log 10) computes for features with differential abundances of bacterial communities in NAFLD patients and controls. NAFLD, patients with non-alcoholic fatty liver disease; unc, unclassified.

Table S1. Expression of markers of Tlr signaling cascade in NAFLD patients and controls.

	C	NAFLD
***MYD88* mRNA expression (% of control)**	100 ± 14.5	117.1 ± 12.7
***TIRAP* mRNA expression (% of control)**	100 ± 15.5	171.8 ± 39.2
***IRF3* mRNA expression (% of control)**	100 ± 14.3	119.9 ± 22.1

Data are shown as means ± SEM, n=10 controls, n=31 NAFLD patients. C, healthy controls; IRF3, interferon regulatory factor 3; MYD88, myeloid differentiation response 88; NAFLD, patients with non-alcoholic fatty liver disease; TIRAP, TIR domain containing adaptor protein.

Table S2. Pearson correlation between the bacterial taxa and expression of *TLR1*.

Genera	Correlation Coefficient	*P value*
o_Clostridiales	-0.081	0.72
g_Streptococcus	-0.121	0.59
g_Ruminococcus2	0.323	0.14
**g_Ruminococcus**	**-0.439***	**0.04**
g_Roseburia	-0.006	0.98
g_Romboutsia	-0.041	0.86
g_Parabacteroides	0.057	0.80
g_Oscillibacter	-0.160	0.48
g_Lactobacillus	0.144	0.52
g_Intestinibacter	-0.192	0.39
**g_Holdemanella**	**0.573***	**0.01**
**g_Gemmiger**	**-0.419***	**0.05**
g_Fusicatenibacter	-0.014	0.95
g_Faecalibacterium	-0.090	0.69
g_Escherichia/Shigella	-0.239	0.28
g_Dorea	0.138	0.54
g_Dialister	-0.337	0.13
g_Coprococcus	-0.154	0.49
g_Collinsella	-0.031	0.89
g_Clostridium.XVIII	-0.081	0.72
g_Clostridium.IV	-0.060	0.79
g_Catenibacterium	0.210	0.35
g_Blautia	-0.120	0.59
g_Bifidobacterium	-0.119	0.60
g_Barnesiella	-0.165	0.46
g_Bacteroides	-0.148	0.51

g_Anaerostipes	-0.162	0.47
g_Alistipes	-0.051	0.82
f_Ruminococcaceae	-0.314	0.15
f_Lachnospiraceae	-0.137	0.54
f_Coriobacteriaceae	-0.001	0.99

n=7 controls, n=16 NAFLD patients, *$p \leq 0.05$. Letters before the name represents the taxonomical level, g=genus, f=family, o=order.

Table S3. Primer sequences used for real-time PCR.

	Forward (5´- 3´)	Reverse (5´- 3´)
	**Human sequences**	
18S	gggcccgaagcgtttacttt	cgccggtccaagaatttcac
IRF3	gaggtgacagccttctaccg	tgcctcacgtagctcatcac
MYD88	gcacatgggcacatacagac	gacatggttaggctccctca
TIRAP	agcccaagaagaggcccaac	tggcacacgcagacgtgata
TLR1	agggtcagctggacttcaga	taattttggatgggcaaagc
TLR2	attgtgcccattgctctttc	ctgcccttgcagataccatt
TLR3	agccttcaacgactgatgct	tttccagagccgtgctaagt
TLR4	tgagcagtcgtgctggtatc	cagggcttttctgagtcgtc
TLR5	gagcccctacaagggaaaac	tgctgatggcattgctaaag
TLR6	caaggccctccaataactca	cattgcaagggaagagggta
TLR7	aaatgtcacagccgtccctac	ttatttttacacggcgcaca
TLR8	tccttcagtcgtcaatgctg	cgtttggggaacttcctgta
TLR9	aaatggcctttgactcatgg	acagcagctacagggaagga
TLR10	actttgcccaccacaatctc	cccagaaaagcccacattta
	**Murine sequences**	
18S	gtaacccgttgaaccccatt	ccatccaatcggtagtagcg
F4/80	tggctgcctccctgactttc	caaggatccctgccctgcact
Tlr1	cgcctggacccagagtttgt	cgcacccaggaaggtcagtt
Tnfα	cagccaaccaggcagcgttcct	cctgccacaagcaggaatga

IRF3, interferon regulatory factor 3; MYD88, myeloid differentiation primary response 88; TIRAP, TIR domain containing adaptor protein; TLR, toll-like receptor; Tnf, tumor necrosis factor.
